# Retraction Note: Carvacrol and PPARγ agonist, pioglitazone, affects inhaled paraquat-induced lung injury in rats

**DOI:** 10.1038/s41598-022-05046-9

**Published:** 2022-01-18

**Authors:** Fatemeh Amin, Arghavan Memarzia, Hamideh Kazemi Rad, Hamid Reza Kazerani, Mohammad Hossein Boskabady

**Affiliations:** 1 Research Institute of Basic Medical Sciences, Rafsanjan University of Medical Sciences, Rafsanjan, Iran; 2 Department of Physiology and Pharmacology, School of Medicine, Rafsanjan University of Medical Sciences, Rafsanjan, Iran; 3 Applied Biomedical Research Center, Mashhad University of Medical Sciences, Mashhad, Iran; 4 Department of Physiology, Faculty of Medicine, Mashhad University of Medical Sciences, Mashhad, Iran; 5 Department of Physiology, Faculty of Veterinary Medicine, Ferdowsi University of Mashhad, Mashhad, Iran

Retraction of: *Scientific Reports* 10.1038/s41598-021-87546-8, published online 14 April 2021

The Editors have retracted this article. After publication, the Editors were informed that several related papers^1–3^ were being prepared or under consideration elsewhere at the time of submission of this article. Some of the conclusions presented in this article are reported in other articles from the same authors^3,4^. In addition, there are inconsistencies between Figs. 1A, 2C, 5A and 7C, and the underlying data. The Editors therefore no longer have confidence that the conclusions presented are adequately supported by the data.

None of the authors agree to this retraction.
